# Assessing the credibility and transferability of the patient compassion model in non-cancer palliative populations

**DOI:** 10.1186/s12904-018-0358-5

**Published:** 2018-09-13

**Authors:** Shane Sinclair, Priya Jaggi, Thomas F. Hack, Susan E. McClement, Shelley Raffin-Bouchal, Pavneet Singh

**Affiliations:** 1 Faculty of Nursing, University of Calgary, 2500 University Drive NW, Calgary, AB T2N 1N4 Canada; 2 Department of Oncology, Cumming School of Medicine, University of Calgary, 2500 University Drive NW, Calgary, AB T2N 1N4 Canada; 3 College of Nursing, Rady Faculty of Health Sciences, University of Manitoba, 89 Curry Place, Winnipeg, MB R3T 2N2 Canada; 4 Research Institute in Oncology, Hematology, Cancer Care, 4005E-675 McDermot, Winnipeg, MB R3E 0V9 Canada; 5 Psychosocial Oncology and Cancer Nursing Research, St. Boniface Hospital Research Centre, Room CR3018, 369 Taché Avenue, Winnipeg, MB R2H 2A6 Canada

**Keywords:** Compassion, Patient compassion model, Credibility, Transferability, Psychometric properties, Qualitative, Grounded theory

## Abstract

**Background:**

A lack of evidence and psychometrically sound measures of compassion necessitated the development of the first known, empirically derived, theoretical Patient Compassion Model (PCM) generated from qualitative interviews with advanced cancer inpatients. We aimed to assess the credibility and transferability of the PCM across diverse palliative populations and settings.

**Methods:**

Semi-structured, audio-recorded qualitative interviews were conducted with 20 patients with life-limiting diagnoses, recruited from 4 settings (acute care, homecare, residential care, and hospice). Participants were first asked to share their understandings and experiences of compassion. They were then presented with an overview of the PCM and asked to determine whether: 1) the model resonated with their understanding and experiences of compassion; 2) the model required any modification(s); 3) they had further insights on the model’s domains and/or themes. Members of the research team analyzed the qualitative data using constant comparative analysis.

**Results:**

Both patients’ personal perspectives of compassion prior to viewing the model and their specific feedback after being provided an overview of the model confirmed the credibility and transferability of the PCM. While new codes were incorporated into the original coding schema, no new domains or themes emerged from this study sample. These additional codes provided a more comprehensive understanding of the nuances within the domains and themes of the PCM that will aid in the generation of items for an ongoing study to develop a patient reported measure of compassion.

**Conclusions:**

A diverse palliative patient population confirmed the credibility and transferability of the PCM within palliative care, extending the rigour and applicability of the PCM that was originally developed within an advanced cancer population. The views of a diverse palliative patient population on compassion helped to validate previous codes and supplement the existing coding schema, informing the development of a guiding framework for the generation of a patient-reported measure of compassion.

## Background

Compassion through the lens of patients with life-limiting health conditions has been described as being both dispositional and relational. It is nonetheless insufficient for healthcare providers (HCPs) to simply possess virtues, as these good and noble qualities need to be coupled with understanding and action, aimed at alleviating the suffering of another person to be considered compassion [1]. These virtuous motivators and orientation toward action distinguishes compassion from sympathy, in that compassion is not a visceral, pity-based response towards a person’s circumstance or misfortune [2–4]. Furthermore, sympathy is a concept that is often coupled with a negative connotation, particularly from patients who find it unhelpful and demoralizing [3, 4]. While bearing a number of similarities with the construct of empathy, compassion requires action and is motivated by virtues such as love, kindness, and genuineness, thereby rendering it more dynamic and distinct [3–6].

Although various tools exist for measuring compassion in healthcare, they often conflate these surrogate constructs with compassion, and lack the rigorous establishment of psychometric properties [7]. A recent comprehensive and critical review identifying and evaluating existing compassionate care measures found a paucity of tools that theoretically conceptualize compassion as a multidimensional construct and perhaps most remarkably failed to incorporate the perspective of the recipients of compassion—patients [7]. This review identified one tool that measured compassion as an emotion in HCPs, failing to account for the crucial, observable dimensions such as behaviour or action, which are among its key defining features [8, 9]. Three tools measured the HCPs ability to provide compassionate care [10–12], and only one measured compassion in relation to the patient’s care experience [13]. Another tool, the Geriatric Attitudes Measure [14], despite being validated, measures limited facets of compassion. Due to the nascent nature of compassion measures and the underutilization of patients’ perspectives, there is a need to develop a patient reported compassion measure based on a patient-informed, theoretical model of compassion.

**Fig. 1:**
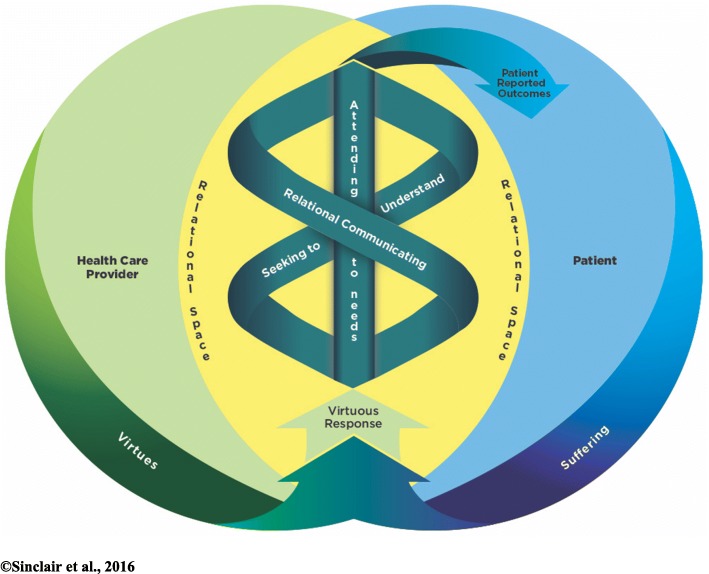
Patient Compassion Model

In recognizing these limitations, a Patient Compassion Model (PCM) (Fig. 1) was developed by our research group from the perspective of advanced cancer inpatients (*n* = 53) using a rigorous grounded theory approach [1]. A complex relationship was outlined between six mutually informing dimensions of the construct of compassion: *virtues,* as the good and noble qualities of HCPs that served as motivators of compassion; *relational space*, highlighting the highly relational nature of compassion where patients initially sensed their HCP capacity for compassion; *virtuous response*, the initial response of a HCP towards the person in suffering; *seeking to understand* the patient as a person and their needs; *relational communicating,* consisting of verbal and non-verbal displays of compassion within the clinical encounter; and *attending to needs,* the quintessential outcome of compassion—action aimed at addressing patients multifactorial suffering [1]. This multi-dimensional compassion construct represents, to our knowledge, the first rigorously established, patient informed, clinically relevant, and empirically derived, theoretical model [1]. While representing a comprehensive model, patients nonetheless recognized fluidity, overlap, and variance across and within the aforementioned dimensions, affirming the personalized nature of expressions (HCPs) and experiences (patients) of compassion, the dynamic nature of compassion, and the constructs adaptability to context and circumstance [1].

In qualitative research, an imperative means of ensuring that the theoretical foundation of a new construct stands on a solid foundation and is ‘trustworthy’ is to determine the credibility and/or transferability of the construct within the same or other patient populations [15–17]. Determining the credibility and transferability of an empirically derived theoretical model addresses two interrelated methodological issues limiting the initial qualitative, and subsequent measure development studies—the lack of follow up studies to verify the results of qualitative studies and the lack of initial content validity with end users within measure development studies, respectively. While qualitative researchers recognize the non-generalizability of their results at the outset of their research projects, and are quick to dismiss criticism from well-intended (yet misguided) quantitative reviewers who suggest otherwise, there is a tendency to treat their findings as *fait accompli* after their study is completed. To determine the extent to which the theory is in fact grounded in other contexts, Grounded Theorists encourage further development of the theory in subsequent studies and different settings, thereby cautioning researchers from taking a *fait accompli* stance to their theory [18]. In doing so, researchers can clarify additional understandings by modifying their initial findings and theoretical model accordingly [15, 18].

An adjacent issue hindering subsequent measure development studies after the theory has been established, is the need to conduct initial content validation to fortify the theoretical foundation that the measure purports to assess. While further steps of validation such as exploratory and confirmatory factor analysis can demonstrate that the questions or ‘items’ of a measure are aligned with researchers’ understandings of a construct, this understanding assumes that the construct is fully comprehensive and accurate. Confirming the content validity of the theory or model can enhance the content validity of a measure directly from those whom the measure will be administered to, as there is “great value of adding [an additional] layer of feedback to the elaboration of a conceptual model before its implementation” (p. 561) [19]. As it’s been argued by others, prior to measure development and its subsequent validation, conducting effective qualitative research is considered a crucial component to ensuring that the instrument possesses adequate content validity and to ensuring fidelity between the proposed measure and its theoretical foundation [20, 21].

The objective of the present study was to assess the credibility and transferability of the PCM among a convenience sample of 20 non-cancer patients living with life-limiting illnesses in multiple healthcare settings, a method of model validation utilized in a previous study [19]. In doing so, we also aimed to establish additional content/face validity for an ongoing study to develop a patient reported compassion measure for palliative patients living with diverse, incurable and life-limiting illnesses.

## Methods

### Study population

A convenience sample of twenty adult patients (> 18 years of age) living with a life-limiting illness was recruited from 4 different care settings (acute care, home care, residential care, and hospice care) within Calgary, Alberta, Canada. This sample size was chosen for the following reasons: 1) our past experience conducting qualitative studies suggested that 20 patients would be sufficient to meet the study aims; 2) data saturation had previously been reached in our original study; and 3) the study’s purpose was to assess the relevancy of the PCM, not to generate a theory or rich description. While we felt that twenty patients would be sufficient, we remained open to increasing recruitment if necessary, an option that based on our rich data did not need to be exercised. To be eligible for the study, potential participants had to be able to speak and read English, be living with an incurable, non-cancer diagnosis, and demonstrate no signs of confusion (as determined by the clinical team). This study was approved by the University of Calgary Conjoint Health Research Ethics Board (REB #16–1460). Written informed consent to participate in the study was obtained from participants prior to conducting the interview. The following demographic information was collected post-interview: care location; age; gender; marital status; highest education level attained; employment status; religious group affiliation; religious and spiritual status; and primary disease type (Table 1).

**Table 1:** Patient Demographics for 20 patients

Characteristic	Frequency (Percentage)
Acute Care	5 (25.0%)
Residential Care (Long Term Care, Seniors Home, Supportive Living Facility)	5 (25.0%)
Hospice Care	5 (25.0%)
Home Care	5(25.0%)
Median Age (Range) (years)	77 (37–99)
Gender:	
Male	9 (45.0%)
Female	11 (55.0%)
Received Care from Following Healthcare Professionals Over the Last Month:	
Physician	19 (95.0%)
RN	19 (95.0%)
LPN	16 (80.0%)
Nursing Assistant	12 (60.0%)
Nurse Practitioner	12 (60.0%)
Social Work	9 (45.0%)
Spiritual Care	7 (35.0%)
Psychology	4 (20.0%)
Physio Therapist	8 (40.0%)
Occupational Therapist	6 (30.0%)
Recreational Therapist	6 (30.0%)
Pharmacist	10 (50.0%)
Religious Group Affiliation:	
Roman Catholic	1 (5.0%)
Protestant	10 (50.0%)
Jewish	1 (5.0%)
Muslim	0 (0.0%)
Buddhist	0 (0.0%)
Other	2 (10.0%)
Other	6 (30.0%)
Spiritual Status:	
Spiritual and religious	9 (45.0%)
Spiritual but not religious	7 (35.0%)
Religious but not spiritual	0 (0.0%)
Neither	4 (20.0%)
Highest Level of Education	
No formal education	0 (0.0%)
No formal education	1 (5.0%)
Some High School	8 (40.0%)
High School- Grade 12 completed	3 (15.0%)
Some University/College or Technical school	3 (15.0%)
University/College or Technical school	4 (20.0%)
University/College or Technical school	1 (5.0%)
Primary Disease Type	
Dementia	4 (20.0%)
Cerebrovascular disease	1 (5.0%)
COPD or other end-stage lung disease	6 (30.0%)
CHF or other end stage heart disease	3 (15.0%)
End stage renal disease	0 (0.0%)
Life-limiting debility from other cause (e.g. chronic infection, MS, ALS)	6 (30.0%) (2 ALS, 1 Arthritis, 1 AIDS, 1Lymphodema, 1 Cystic Fibrosis
Marital Status	
Never married	3 (15.0%)
Married	6 (30.0%)
Common Law/Cohabitating	1 (5.0%)
Divorced	3 (15.0%)
Separated	0 (0.0%)
Widow(er)	7 (35.0%)

### Data collection and analysis

For this study, we adapted the semi-structured interview guide from our previous study of 53 cancer patients, which informed the development of the PCM (Table 2) [1]. Face-to-face interviews were conducted with participants by an experienced qualitative interviewer at a mutually agreeable time. After patients were provided the opportunity to share their perspectives and experiences of compassion directly, each participant was provided with a detailed overview of the PCM and asked to assess similarities and differences. Participants were encouraged to draw upon their personal understanding of compassion in considering the remaining interview questions that focused on specific facets of the PCM in order to focus on issues of congruence (or lack thereof) between their understandings and the model, versus their general agreement with the model (Fig. 1). Participants were given opportunity to speak to the relevance of the model as a whole and its associated dimensions, and were encouraged to suggest modifications (e.g. addition or removal of categories/themes) (Table 2). All interviews were audio-recorded, transcribed verbatim, and were then independently verified by a professional transcriptionist and a research assistant. Interviews took place from October – December 2016 and analysis occurred concurrently throughout, ending in January 2017. The interviews averaged 1.0–1.5 h and were conducted in the residences of home care patients and within private spaces for patients living in healthcare facilities. Four members of the research team (T.H., S.M., S.R.B., S.S) used constant comparative analysis to analyze the interview transcripts. This involved comparing the codes from each subsequent transcript with previous codes from earlier interview transcripts within this study as well as the pre-existing codes contained within our coding schema from the previous study of advanced cancer patients [1]. The initial coding schema contained over 600 individual codes that were used in coding the current transcripts, with additional codes emerging from the interviews being added. Transcripts were first coded independently in a line-by-line manner by each member of the analysis team and then collectively coded line-by-line until consensus was achieved. The final stage of analysis occurred at a higher theoretical level, whereby the key components and PCM were modified and/or verified after all transcripts had been analyzed. While this occurred in an on-going, iterative manner throughout the study, this was finalized via two 3-h videoconferences at the end of the study.

**Table 2:** Interview guiding questions

1. In terms of your own illness experience, what does compassion mean to you? Can you give me an example of when you experienced care that was compassionate?	
2. Having seen and being provided with an overview of the compassion model, in general how do you feel it relates to personal understanding and illness experience? [Do you feel anything is missing? Do you feel anything needs to be removed?]	
3. Considering your ethnic background, is there anything you would consider changing related to the compassion model?	
4. What do you consider the key qualities of a compassionate health care professional?	
5. How can you tell if a healthcare provider genuinely wants to understand you as a person? [How would you know a healthcare provider is seeking to understand you?; What does a healthcare provider do to make you feel understood as a person?]	
6. When a healthcare provider is interacting with you how do you know that they are providing compassionate care?	
7. Thinking about the first time that you met a healthcare provider, what is it about them that tells you they are compassionate? [How can you tell you are receiving compassion based on your healthcare providers’ initial response to you?]	
8. What are the things that a healthcare provider does [their actions] that make you feel you are receiving compassionate care?	
9. Is there anything that we have not talked about today, that we have missed or you were hoping to talk about today?	

## Results

The six key categories of the PCM were verified, and no new categories or themes were generated from the data. Several additional, unique codes within the pre-established categories and provided further insight and additional potential items to aid in the development of a compassion measure. Personal experiences of compassion shared by patients traversed the core categories of the original model and are substantiated below with patient exemplars [1].

### Category: virtues

Patients identified the innate virtues of their HCP(s) as a key driver(s) of compassion. In particular, they identified qualities of *kindness, love, understanding, acceptance, genuineness, honesty, and sincerity* as imperative*.* These noble qualities were identified previously and further underscored by current participants as core necessities in generating and imbuing compassion. Within this patient cohort, an additional virtue, *wisdom,* was identified as being essential in providing compassion.

If you aren’t kind, maybe you’re not motivated to be compassionate. Yeah, it seems like that would be a fundamental requirement (Patient 15).

You’d want them to have acceptance and you’d want them to have patience, you’d want them to have understanding (Patient 11).

Well, one [key quality of a compassionate healthcare provider] is wisdom. Wisdom as in using their knowledge to help others or even if they don’t know, to learn. For them to continue to grow (Patient 11).

### Category: relational space

Patients described compassion as a highly relational construct, occurring between a responder and a recipient. Relational space, according to patients, extended beyond a mere clinical relationship in that it required HCPs to actively know and be known by the patient, and engage in their suffering. Patients described a deep connection between themselves and their HCP as a result of entering into this relational space that went beyond a healthcare provider-patient relationship. While this realm was largely experienced intuitively by patients, it also was tangibly evident in the degree to which caregivers were engaged with the patient when interacting with them.

#### Theme: patient awareness

You know there’s compassion and you know if there isn’t sometimes (Patient 15).

Some people within minutes you can tell. Instinctively you know that they’re here to help you (Patient 3).

You get --- I get a feeling, a nice feeling…like he gives me a good vibe, a very strong vibe (Patient 10).

You can feel it. You can see it. You know there’s something; there’s a connection (Patient 11).

#### Theme: engaged caregiving

But I want to know about people. It’s not enough that they’re there for pretty intimate parts of my life. So if that’s the case, I want to get to know them. So it’s not just a one-way thing with health practitioner to patient, but it’s important the other way. So for somebody to recognize it’s important that I know you too, right? (Patient 15).

And then I think that’s one of the things that make them compassionate and the same thing, we think of the doctors as old and you never ask them about their families and yet the family doctor now likes to talk about his family too (Patient 2).

### Category: virtuous response

Virtues were not understood by patients as simply traits or dormant qualities, but rather they needed to generate a virtuous response in relation to compassion. The centrality of virtues was endorsed by all participants, with one participant suggesting that virtues actually needed to be more prominently embedded across the entire model. Study participants felt that the virtuous nature of this response, in comparison to an emotional or duty-based response, attested to the largely unconditional nature of compassion that wasn’t based on a pre-existing criteria or relationship. Participants reported that HCPs virtues were externalized by: approaching the patient as a person; making the person the priority in clinical encounters and decision making; and by having their patients best interest at heart. Examples included a HCP taking a genuine interest in getting to know a person at an initial visit or in advocating with a patient’s insurance company for extended drug coverage.

#### Theme: knowing the person

Like one knew us for many years, but the other two we had just met them and they were leaving and they were hugging us and it’s like you can feel that instant bond with them right? (Patient 18).

Compassion means that somebody cares what I’m going through (Patient 15).

#### Theme: person as a priority

To me someone who is compassionate is he’s to do his duty fully (Patient 16).

Well…some people within minutes you can tell. Instinctively you know that they’re here to help you and others you know that they’re just there waiting for the pay package on Friday (Patient 3).

#### Theme: beneficence

Your everyday life and your everyday struggles and like they came in and like did you guys get your furnace fixed? Like they’re all compassionate about not just his medical, but our lives, our everyday struggles (Patient 18).

Well, people that are concerned about you, and they look after you, or help you when they can (Patient 17).

### Category: seeking to understand

Patients in this study highly endorsed the category of seeking to understand the person and their individualized needs. While addressing their medical needs were paramount, patients identified compassionate HCPs as those who did not medicalize the person or base their understanding of the patient solely on the patient’s medical record. In contrast to an over-reliance on the medical record, patients felt that a key facet of compassion was a desire to understand them on a personal level and taking this into account when considering their health issues, and asking them questions about themselves and their health concerns concurrently.

#### Theme: seeking to understand the person

I want you to look at me as a person and not all the issues that I have (Patient 19).

You can’t be compassionate if you just read everything off a piece of paper you know? You didn’t see me when he’s going through that paper. (Patient 10).

#### Theme: seeking to understand the person’s needs

It’s the way [the doctor] approaches you and trying to understand – he’s trying to understand what you want (Patient 4).

They’re prepared to --- they’re ready to hear what you need (Patient 11).

…they could hear my pain… just like when a baby is crying, you can tell if it like bumped its elbow or if it fell off the top of the dryer. Like you can tell the difference…it’s all your five senses (Patient 11).

### Category: relational communicating

The previously identified themes within the category of relational communicating of Affect, Behaviour, Engagement, and Demeanor, were also endorsed to varying degrees in this study, acknowledging that there are a number of indicators of compassion that seem to be specific to communication. Whether conveyed through their demeanor, displays of emotion, active listening, or the tenor in which information was provided, participants felt that compassion was readily identifiable when HCPs communicated in a personalized manner—delivering information in a way that was best understood or received by the person. While each of these themes were broadly endorsed by patients, they emphasized emotional resonance within the theme of Affect, and active listening within the theme of Behaviour as being particularly important to compassion*.*

#### Theme: demeanor

That the attention through your eyes, through your attention alone, you would be acknowledging interest in what the problem is (Patient 9).

You’re looking at them more as eye-to-eye and not breaking your neck. Yeah, I notice more of them today squat to their patient, be it in a chair or in a bed (Patient 2).

Well sometimes you can tell like… tone of voice…eye contact… like I would call it like being present (Patient 15).

#### Theme: affect

How I feel to her and her to me and she can accept the fact that okay (Patient 10).

If you’re telling a sad story and their eyes start to water up… that’s how I tell (Patient 6).

#### Theme: behaviours

The way they speak to you. That is the most important thing. As if they are listening and hearing you (Patient 7).

They tried to really hear what you were saying (Patient 5).

#### Theme: engagement

They’ll put it in your terms. They’ll simplify it by a diagram and give you something that you would understand and break it down to this is where you are, this is where we want to get you (Patient 11).

They come up to the bed and a lot of the nurses will grab your hand or just stand there and talk to you about your life or their life (Patient 5).

Compassion isn’t necessarily being a cheerleader or saying it will all be okay. Just think positively. Compassion is also I think meeting the person where they’re at and not everybody needs or wants to be cheered up right now. (Patient 15).

### Category: attending to needs

The capstone or ultimate outcome of the various facets of compassion was action. The action-orientated nature of preceding categories, culminated in tangible acts to alleviate the suffering of the patient by being readily available and responding proactively when needs arise. While patients felt that compassion-based action needed to be coalesced in routine care, incidences where HCPs went ‘above and beyond’ their job were referenced as some of the clearest indicators of a compassionate HCP.

#### Theme: compassion related needs

Compassion means the people are assisting me to relieve the pain that I have (Patient 3).

But the guy put the needle in and they don’t find blood, so they wiggle it around like an oar until they find something and then jam it in a little deeper… they’ve shown no compassion (Patient 3).

#### Theme: timely

She [home care nurse] does something about it. She’s already got in touch with the doctor because I had a bit of trouble this week (Patient 8).

They are quick to respond and they surround you, so that’s compassion (Patient 11).

#### Theme: action

It wasn’t an easy thing to do, but she [physician] arranged it and she knew that that would really brighten my day and it did (Patient 15).

Even the paramedics, the firemen, they all know me. They come over and shovel the [snow on my] driveway…I have like four guys that come on their own time. Now that’s out of compassion (Patient 18).

## Discussion

The PCM was derived using a rigorous Grounded Theory approach, a large qualitative sample, and multiple stages of data analysis until data saturation was achieved [1]. This produced the first clinically relevant, patient-informed, empirically derived theoretical model of compassion. One of the limitations of the PCM was that it was developed exclusively with advanced cancer patients, limiting its generalizability to other palliative populations. The current study aimed to address this gap by assessing the credibility and transferability of the PCM with non-cancer patients and to further establish content/face validity in developing a patient reported compassion measure among patients living with various life-limiting, incurable illnesses. After analyzing the data, it was determined that our previously established, patient informed definition of compassion was verified within this diverse patient population --“a virtuous response that seeks to address the suffering and needs of a person through relational understanding and action” (p. 195) [1].

Results revealed that the model not only resonated with our patient sample, but the core domains and themes within it were endorsed, suggesting that the overarching concept, structure, and associated dimensions of the model was credible and transferable within other patient populations. Patient participants provided further insight and experiences of compassion, which were incorporated into the pre-existing coding schema, thereby enhancing its comprehensiveness and providing a more robust item pool for measure development. One example was the identification of intention as an additional theme not previously reported [1], which Vachon (2016) identified as a distinguishing feature and primer of compassion that may be influenced by attachment style in caregivers [22, 23], stressing the importance of healthcare provider self-awareness, that may benefit from further research. Another salient example was the identification of an additional virtue, *wisdom,* by a number of study participants, suggesting that compassion involves discernment and not simply an unconditional response. In the virtue ethics literature, *phronesis* or practical wisdom is particularly important within healthcare, which has been defined as “the capacity, in a given set of circumstances to discern what moral choice or course of action is most conducive to the good of the [patient] or the activity in which the [patient] is engaged. *Phronesis* is the intellectual virtue that disposes us habitually to obtain truth for the sake of action” (p. 84) [24]. This highlights the importance and necessity of cultivating the requisite innate qualities to engender a compassionate response.

While the majority of our results were confirmatory in nature, this study also addressed a common limitation of qualitative research--the lack of follow-up studies to substantiate findings and address issues of generalizability. As Glaser and Strauss, the founders of Grounded Theory, suggest “His [the grounded theorist] sociological perspective is never finished, not even when he writes the last line of his monograph—not even after he publishes it, since thereafter he finds himself elaborating and amending his theory…” [18]. After developing a theoretical model, researchers often treat them as a *fait accompli*, defending against alternate interpretations and identified limitations by citing the original study, supporting literature, and their own expertise, rather than returning to the population of interest directly [19]. While the PCM was developed in a rigorous and iterative fashion (verifying the emerging model in subsequent interviews with cancer patients), we felt compelled to address this limitation by including an additional cohort of patient perspectives within diverse disease populations and care settings to determine if understandings and experiences of compassion varied. In using constant comparative analysis, we were able to compare and contrast the unique experiences of 20 patients with life-limiting illnesses with our previous patient sample, further fortifying our theoretical model in the process, in accordance with other studies validating a conceptual model with stakeholders [19]. As such, while further research to assess the credibility and transferability in clinical practice is needed, including integrating clinicians’ perspectives, we feel that the PCM can serve as a clinical and an empirically derived theoretical framework to inform practice, education, and research. While this study suggests that the construct and key domains of compassion are universal, the additional unique codes generated in this study in addition to the 600 individual codes generated in the previous study, emphasize that there is variance in individuals’ understandings and experiences within these collective domains and themes.

As the purpose of this study was to determine the credibility and transferability of the previously developed PCM [1], we felt it was imperative to provide participants with a copy and description of the PCM to obtain direct, informed feedback. While this was a strength of this study, this was also a limitation in that it may have introduced response bias. While we attempted to control against this limitation by: using an interviewer who was neither a member of the clinical nor research team; asking patients to first provide their understanding of compassion prior to being exposed to the model; actively encouraging patients to critique the model; using the constant comparative technique in data analysis to compare and contrast patients’ personal understanding with their subsequent responses related to the model specifically, this may have nonetheless impacted the results. Although we felt that we adequately controlled against this limitation, we did not anticipate the level of congruence and enthusiasm that patients expressed between their own understandings of compassion, and the domains and themes of the PCM. We had anticipated that the majority of categories and themes would resonate with study participants, but did not expect that all of these components would be endorsed—which is likely due to the large qualitative sample size and rigorous process of analysis in our original study [1]. Although we are confident in the transferability and credibility of the PCM, the perspectives of patients represent only one side of the compassion relationship, requiring further research on the perspectives of healthcare providers on this dynamic and relational care construct within clinical care.

In addition to having diverse illnesses and care settings (Table 1), our palliative patient population also came from a cross-section of spiritual and educational backgrounds. While there were a significant number of patients that identified their religious affiliation as Protestant (50%) or had no religious affiliation (30%), in terms of spiritual background, 45% identified themselves as spiritual and religious, and 35% as spiritual but not religious, thereby reflecting a growing trend toward individuals’ primary identification with spirituality, with religious affiliation taking an important, but seemingly secondary role [25]. A further limitation to our study is that while ethnicity was collected as a demographic variable, we are unable to appropriately categorize the various responses and multiple terms utilized by our patient sample to describe their ethnic backgrounds. This limitation suggests that a more distinct and standardized response scale should be utilized for patients to select their ethnic background(s) from. Our current study was however, diverse in terms of our participants’ education level, with 60% of our population having no greater than a high school education, addressing a limitation of our original study that consisted of a highly educated sample, which may have produced an overly intellectual conceptualization of compassion [1].

## Conclusions

The PCM, its six core categories and associated themes, was transferable to non-cancer patients living with an incurable illness, as assessed by patients directly. In addition to providing educators and clinicians with a broadly applicable model of compassion, these confirmatory results provide an empirical foundation for researchers studying compassion within healthcare, including our research teams current study focussed on developing a patient reported experience measure of compassion. In regards to this ongoing study, in addition to addressing an important, but often overlooked stage of content validity [20], the additional codes and nuances of compassion provided by patients within this study will aid in the generation of a comprehensive item pool and ultimately a psychometrically sound measure of compassion to enhance clinical practice, research, and education.

## Abbreviations

HCPs: Healthcare Providers
PCM: Patient Compassion Model
